# Development and Application of Immunoaffinity Column Purification and Ultrahigh Performance Liquid Chromatography-Tandem Mass Spectrometry for Determination of Domoic Acid in Shellfish

**DOI:** 10.3390/toxins11020083

**Published:** 2019-02-01

**Authors:** Si Chen, Xiaojun Zhang, Zhongyong Yan, Yangyang Hu, Yibo Lu

**Affiliations:** 1 Laboratory of aquatic product processing and quality safety, Marine Fisheries Research Institute of Zhejiang Province, Zhoushan 316100, Zhejiang, China; sichen_ns@zjou.edu.cn (S.C.); yanzhongyong@zjou.edu.cn (Z.Y.); 2 Marine and Fisheries Research Institute, Zhejiang Ocean University, Zhoushan 316000, Zhejiang, China; 3 School of Food and Pharmacy, Zhejiang Ocean University, Zhoushan 316022, Zhejiang, China; yangyangHU6@hotmail.com; 4 Jiangsu Meizheng Biotechnology Company Limited, Wuxi 214135, Jiangsu, China; luyibo_byl@163.com

**Keywords:** domoic acid, immunoaffinity column, purification, ultrahigh high performance liquid chromatography tandem mass spectrometry, shellfish

## Abstract

Domoic acid (DA) is a neurotoxin associated with amnesic shellfish poisoning (ASP). Though LC coupled to tandem mass spectrometry (LC-MS/MS) has become the preferred method for DA determination, traditional sample pretreatment is still labor-intensive. In this study, a simple, efficient and selective method for LC-MS/MS analysis of DA in shellfish was established by optimizing clean-up procedures on a self-assembly immunoaffinity column (IAC). Shellfish was extracted with 75% methanol twice and diluted with phosphate buffered saline (PBS, 1:2). The mixture was purified on IAC as follows: preconditioned with PBS, loaded with sample, washed by 50% MeOH, and eluted with MeOH containing 2% ammonium hydroxide. Concentrated analyte was monitored by multiple reaction monitoring (MRM) using electrospray (ESI) positive ion mode throughout the LC gradient elution. Based on the post-extraction addition method, matrix effects for various shellfish matrices were found to be less than 8%. The developed method was fully validated by choosing mussel as the representative matrix. The method had a limit of detection (LOD) of 0.02 µg·g^−1^, showed excellent linear correlation in the range of 0.05–40 µg·g^−1^, and obtained ideal recoveries (91–94%), intra-day RSDs (6–8%) and inter-day RSDs (3–6%). The method was successfully applied to DA determination in 59 shellfish samples, with a detection rate of 10% and contaminated content of 0.1–14.9 µg·g^−1^.

## 1. Introduction

**Figure 1:**
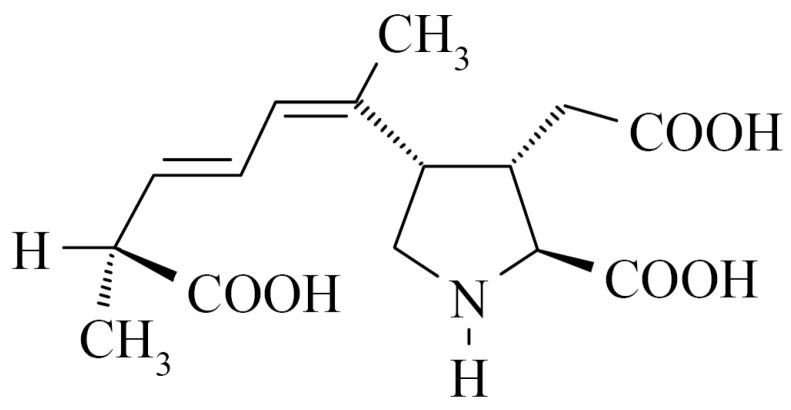
Chemical structure of domoic acid.

Domoic acid (DA) is a glutamic acid analogue neurotoxin associated with amnesic shellfish poisoning (ASP) (Figure 1) [1]. The toxin was identified as the causative agent of the 1987 human poisoning incident in Canada, which occurred after consumption of contaminated blue mussels [2]. The human intoxication syndrome of ASP is characterized by gastrointestinal symptoms of vomiting, abdominal cramp, diarrhea, and neurological symptoms of headache, short-term memory loss, brain damage, and in the most severe cases, death [3]. DA is naturally produced by different species of *Pseudo-nitzschia* and other marine organisms such as red alga *Chondria armata*, then potentially accumulated in shellfish and other crustaceans through the food chain [4,5]. Reports of DA poisoning in wild animals, as well as DA contamination in coastal water have been published throughout the world [5,6,7,8]. To protect human health, Canada, European Union and United States have established that the upper limit of DA in the wet tissue of shellfish samples should not exceed 20 µg·g^−1^ [9].

Currently, methods for DA identification and quantification include toxicity assay in mouse [10], enzyme-linked immunosorbent assay (ELISA) [11,12], thin layer chromatography (TLC) [13] and liquid chromatography (LC) [8,14,15,16,17,18,19]. The standard AOAC method, LC coupled with ultraviolet detection (LC-UV), has been widely employed and modified for DA monitoring, yet false positive results can occur when the samples contain interfering species [15]. Several LC coupled with fluorescence detector (LC-FLD) methods were proposed for DA determination in plankton and seawater through tedious derivatization procedures, but only two of these procedures have been successfully applied to shellfish matrices [16,17]. Due to its high sensitivity and selectivity, LC coupled to tandem mass spectrometry (LC-MS/MS) has become the preferred method for the confirmatory analysis of DA in shellfish. Possible matrix effects of shellfish make an efficient pretreatment procedure necessary before MS detection. Solid-phase extraction (SPE) based on strong anion exchange (SAX) has been employed as the most common clean-up method for biological samples [9,18,20]. Considering the fact that the practical operation of SAX could be labor-intensive, an immunoaffinity column (IAC) [21], molecularly imprinted solid-phase extraction (MISPE) [9], magnetic solid-phase extraction (MSPE) [22] and other SPE deformations are emerging as promising and innovative sample preparation techniques due to their specificity and convenience.

IAC clean-up utilizes the high specificity of imprinted antibodies to extract or concentrate target compounds from complex matrices [23]. With the strong advantages of reducing both interfering background and extraction time, this efficient and consistent technique has been widely applied as the alternative pretreatment method for routine SPE procedure to detect mycotoxins, veterinary drugs, pesticides and vitamins in food [24], yet only a few studies have been devoted to develop specific IAC to extract phycotoxins from marine organism tissue. Kawatsu et al were the first to report DA confirmation in Japanese mussels by LC coupled with IAC using an anti-DA monoclonal antibody as a ligand [21]. However, the proposed IAC seemed to be only compatible with phosphate-buffered saline (PBS) but organic solvents, and detailed information on method validation was lacked for possible further application. A tetrodotoxin-specific IAC was successfully developed and commercialized by our laboratory for LC-MS/MS determination of the potent neurotoxin in various marine organisms [25]. The aim of this work was to develop self-assembled IAC and corresponding procedures for DA purification from shellfish samples, which would be compatible with the extensively used methanolic extract and later analytical step of LC-MS/MS. The method was validated in terms of sensitivity, linearity, precision and repeatability by using mussel as the representative matrix, and then applied to DA determination in shellfish samples collected from local markets and culturing farms.

## 2. Results and Discussion

### 2.1. Optimization of UHPLC-MS/MS Conditions

Various DA isomers often co-occur at variable low levels in natural shellfish samples [26]. It is common practice to report the sum of DA and its major isomer C5’-*epi*-domoic acid (*epi*-DA) as the quantitative results of DA analysis [27]. Given the fact that DA exhibits higher toxicity than the isomers, and DA shares identical molecular weight and fragment ions with its isomers, selective extraction or complete resolution is necessary to avoid any potential overestimation during the LC-MS/MS analysis. In this study, IAC specific to the DA but not its isomers was developed and employed as the clean-up strategy, thereby simplifying subsequent instrumental analysis. For chromatographic separation, the mobile phase of acetonitrile/water and additive of formic acid/ammonium acetate were chosen. An LC gradient elution on an ACQUITY UPLC BEH C18 column with a high amount of organic component was utilized to obtain the best peak shape for the target analyte.

**Figure 2:**
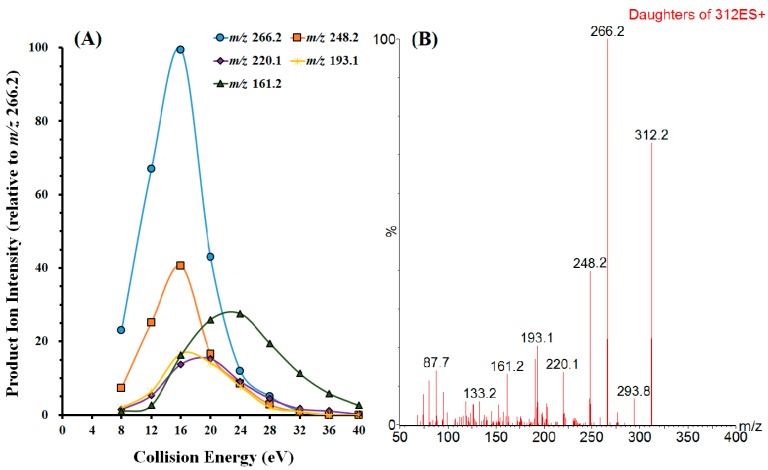
(**A**) The influence of collision energy on the product ion intensity and (**B**) Product ion MS2 spectra of [M + H]^+^ ion (*m*/*z* 312.2) at a collision energy of 16 eV.

To obtain maximum abundance of molecular ions and generate higher sensitivity for analyte, optimization of MS/MS parameters was necessary. By direct infusing a DA standard solution (5.0 μg·mL^−1^) at a flow rate of 5 μL·min^−1^, a characteristic protonated adduct was obtained under full scan mode. The predominant peak corresponding to the [M + H]^+^ ion at *m*/*z* 312.2 was chosen as the precursor ion. Collision energy was then introduced to generate product ion spectra of the selected [M + H]^+^ ion using product ion scan mode for DA, and fragment ions at *m*/*z* 266.2 ([M + H-HCOOH]^+^), *m*/*z* 248.2 ([M + H-HCOOH-H_2_O]^+^), *m*/*z* 161.2 ([M + H-HCOOH-C_2_H_3_O_2_N-H_2_O-CH_2_]^+^), *m*/*z* 220.1 ([M + H–2HCOOH]^+^ ), *m*/*z* 193.1 ([M + H–HCOOH–C_2_H_3_O_2_N]^+^) were observed as the major and consistent ones. In order to optimize the MRM conditions for DA detection, product ion spectra were acquired at collision energies ranging from 8 eV to 40 eV at 4 eV intervals using optimized source conditions (Figure 2). Though fragment ion *m*/*z* 161.2 is less abundant but more specific, no significant difference was found in the MRM interference or background among transitions 312.2 > 161.2, 312.2 > 266.2 and 312.2 > 248.2. The most abundant and second most abundant fragment ions at *m*/*z* 266.2 and *m*/*z* 248.2 were selected for quantification purpose and qualitative confirmation, respectively.

### 2.2. Optimization of Extracting Conditions

The most extensively used extraction procedures for DA involve 0.1 M HCl or aqueous methanol (MeOH) [28,29]. Previous studies suggested that low pH would induce the decomposition of DA [30,31], while extraction with 50% MeOH could provide a complete recovery of DA, as well as the other co-extracting yet interfering components from the sample matrix [29]. Higher percentages of organic solvent in the extraction system usually guarantee effective protein precipitation and lipid removal, which potentially enhance the column reusability by addressing the problem of sorbent clocking or blocking. To investigate the influences of the MeOH proportion and number of extractions on the DA recoveries, experiments were carried out by spiking DA-free shellfish samples at a level of 0.5 µg·g^−1^ before the extraction. The extracts were then purified with the IAC and analyzed by the UHPLC-MS/MS system. As shown in Table 1, single extraction with 50% or 75% MeOH was more effective than 90% MeOH for the DA recovery, exhibiting 11% and 17% higher efficacy, respectively. When the number of extractions increased from one to two, DA recovery for all the extractives exhibited an increase of 10–17%, and a similar changing trend was noticed among the 50%, 75% and 90% MeOH treated samples. Since the 50% methanolic extract of shellfish was relatively cloudier, which subsequently extended the sample loading process on IAC, DA extraction was performed twice with 75% MeOH in a subsequent study.

### 2.3. Preparation of the IAC Column

**Figure 3:**
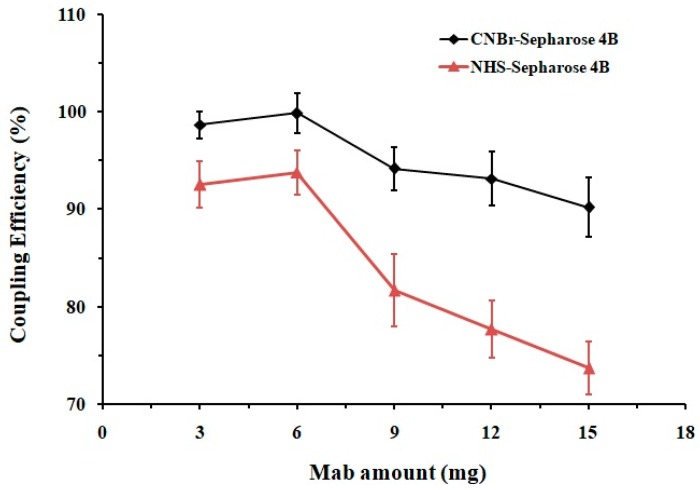
Coupling efficiency of CNBr- and NHS-activated Sepharose 4B (1 mL) conjugated with different amount of mAb (*n* = 3).

The antibody specificity and column capacity are important factors that influence analyte recovery during the IAC-based purification process. In this study, DA-BSA (bovine serum albumin) conjugate was synthesized, and monoclonal antibody (Mab) against DA was successfully produced utilizing the hybridoma method [32]. Sepharose 4B was chosen as the ideal support matrix due to the following characteristics: it is water-insoluble but hydrophilic, can be easily activated, and has good physicochemical stability, high specificity and adsorption capacity. Both CNBr and NHS activated Sepharose 4B were employed for the antibody-matrix coupling to compare their efficiency. Different amounts of Mab were separately conjugated with these two matrices while the protein concentrations before and after the reactions were measured. As shown in Figure 3, when treated with the same amount of Mab (3–15 mg for 1 mL·gel), the coupling capacity of the CNBr-Sepharose 4B was 6–17% higher than that of the NHS-matrix. Conjugation of Sepharose 4B matrices with 6 mg Mab reached chemical saturation, among which the CNBr-activated one exhibited the highest coupling efficiency of 99 ± 2%. An excessive amount of antibody (>6 mg) might provide an increased blocking effect on the matrix surface, potentially reducing antigen-antibody binding and column capacity; therefore, 6 mg of Mab for per mL CNBr-Sepharose 4B was recommended for the chemical reaction in order to prevent ligand waste and maintain the column performance.

### 2.4. Characteristics of the IAC Column

**Figure 4:**
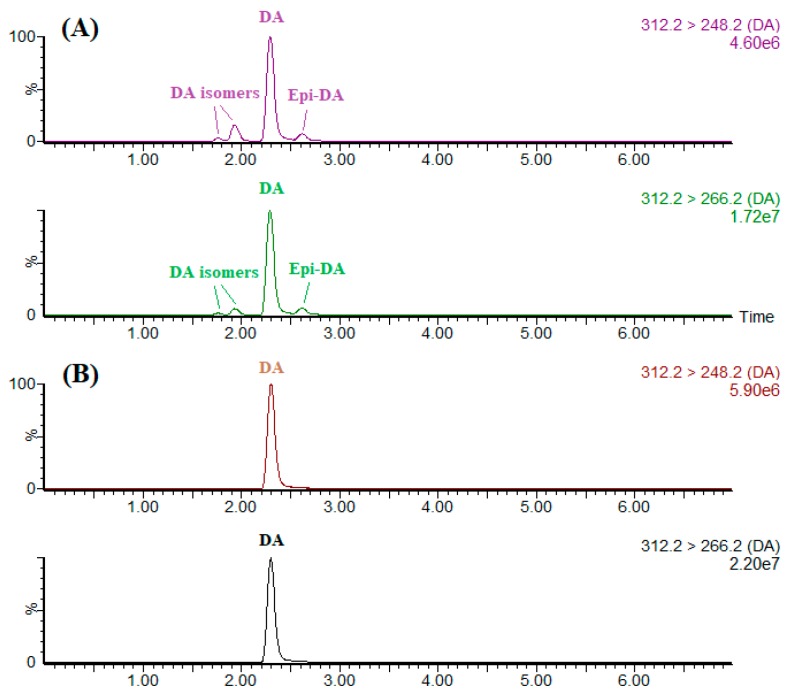
MRM analyses for DA and its isomers in 1-mL portions of CRM-ASP-Mus-d extract (**A**) before and (**B**) after IAC treatment (injection volume, 2 μL).

Employing the high specificity of IAC as a sample pretreatment for LC-MS/MS offers a significant advantage over the existing methodology by providing the direct quantitative information of DA itself. CRM-ASP-Mus-d is a thermally sterilized homogenate of mussel tissue (*Mytilus edulis*) contaminated with DA and some of its isomers. Portions (1 mL) of methanolic extract were either directly evaporated by nitrogen or further cleaned up by IAC. These residues were then reconstituted by 1 mL of the initial mobile phase before the LC-MS/MS analysis (Figure 4). To separate DA from its isomers, a gradient cycle 7 min long was implemented with a 3-min isocratic segment (90% mobile phase B), a 0.5-min linear gradient to 10% B, a 1-min hold, a 0.5-min linear gradient to 90% B and a 2-min hold for column equilibration. As demonstrated by Figure 4, IAC assembled in this study permitted selective extraction of DA from its isomers and other interfering co-extracts in the shellfish sample, eliminating any possibility of overestimation or underestimation of the DA concentration. Studies have showed that DA can transform into *epi*-DA and other iso-DAs through heating, exposure to ultraviolet light or during long-term storage [33]. Since DA was solely extracted and detected, the IAC-based method therefore should preferably be used on fresh shellfish products as within these products, epimerization did not occur.

**Figure 5:**
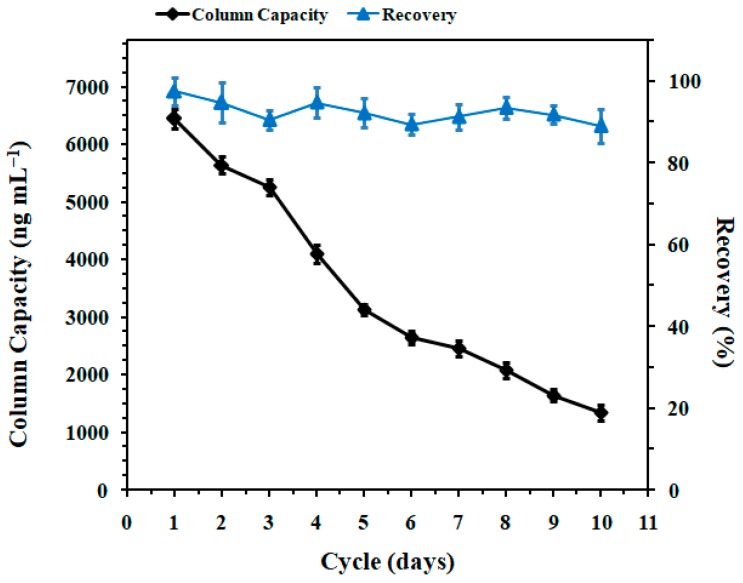
Evaluation on the capacity and recovery of the IAC column (*n* = 3).

Column capacity and reusability depended on the concentration and activity of the immobilized Mab, as well as the chemical stability of the support material. The capacity was evaluated as mentioned in Section 4.4 and presented as micrograms of analyte relative to IAC bed volume (µg·mL^−1^). The column capacity of new IAC was found to be 6.4 µg·mL^−1^, which was sufficient for DA determination and suitable for shellfish products. Each time after DA purification, the IAC was washed and stored in PBS at 4 °C, and the column reusability was evaluated over 10 days at 1-day intervals. For the daily experiment, 1-mL portions of methanolic shellfish extract containing 50 ng DA were diluted with 2 mL PBS and then cleaned up with the optimized IAC procedure. As shown in Figure 5, though the recovery of IAC was consistent in the range of 89–97%, the column capacity decreased when the cycles of usage increased, especially during the third and fourth runs. Once the usage cycle exceeded a value of five, the IAC capacity dropped to 3.1 µg·mL^−1^. Considering the fact that the applicable linear curve was constructed with a concentration of 0.005–4 µg·mL^−1^ in this study, the IAC column therefore has the potential to be reused four times with column capacity above 4 µg·mL^−1^ to meet the method measure range.

### 2.5. Optimization of IAC Loading Conditions

Since organic solvent could denature the antibody and interfere with the antibody-antigen interaction, the primary task in this study was to reduce the MeOH concentration to an ideal level that allowed the analyte to be enriched by IAC without compromising the immunosorbent’s performance. MeOH-PBS mixtures with different volumetric ratios at 1:9, 1:3 and 1:1 were tested as the loading solution. Specifically, 3 mL of each loading solution containing 50 ng DA was applied to the IAC at a constant flow rate of 2 mL·min^−1^. The DA recoveries were found to be close to 95% when the MeOH percentage ranged from 10% to 25%, then showed an obvious decline as MeOH increased to 50% by volume. This result was in accordance with our previous work [25]. To achieve the best recovery and save pretreatment time, the MeOH amount of the sample extract was therefore diluted with PBS to 25% throughout the study.

### 2.6. Optimization of IAC Eluting Conditions

For the convenience of the later LC-MS/MS analysis, a small volume of organic solvent would be preferred to elute the analyte from the IAC. MeOH (10 mL) was initially tested by collecting each 1 mL eluate fraction. However, the results were not satisfactory, with DA recoveries of only 26% and 53% for the first and the second 5 mL eluate, respectively, which indicate that pure MeOH was not strong enough to break the antibody-antigen binding.

Considering the fact that further disruption of the binding interaction could be realized by raising or lowering the pH of the eluting solvent, and DA can decompose under low pH conditions [30,31], acidic (1% formic acid in MeOH, 5 mL) and alkali MeOH (1% and 2% ammonium hydroxide in MeOH, respectively, 5 mL) were selected as the eluant. For each eluting condition, the corresponding eluate fractions were collected and prepared by the same procedure mentioned above. As shown in Table 2, 2% ammonium hydroxide in MeOH gave the best recovery of 95%, and a volume of 3 mL was sufficient to elute the adsorbed DA completely from the IAC. Consequently, 3 mL of 2% ammonium hydroxide in MeOH was selected for the elution condition.

### 2.7. Optimization of IAC Washing Condition

Shellfish matrices are complex and differ in composition from one sample to another; a significant matrix effect and signal suppression were noticed during MS quantitation for DA in various shellfish extracts. Sample amount reduction, optimized sample preparation, and isotope internal standard or matrix-matched standards are usually used to compensate for these effects [34]. In this study, we aim to further remove the interfering substances from shellfish matrices by washing the IAC after the loading step to improve the accuracy of DA quantitation. Various aqueous MeOH percentages (10%, 20%, 30%, 40%, 50%, 60%, 70%, 80%, *v*/*v*) were selected and compared as the washing solvent. To investigate whether the MeOH concentration would interfere with the antibody-antigen bound, experiments were initially carried out by loading 3 mL of an MeOH-PBS (1:3, *v*/*v*) solution containing 50 ng DA into the IAC, followed by rinsing with 6 mL of the washing solvent mentioned above. The results showed that the DA recovery was still higher than 91% when the MeOH concentration in washing medium reached 50%, then underwent nearly 18% and over 80% decrease once the MeOH level exceeded 60% and 70%, respectively.

To test the efficiency and consistency of IAC in eliminating nonspecifically bound compounds from the shellfish matrices, additional experiments were carried out with methanolic extract of blank shellfish samples that were spiked with the same amount of DA standard. Portions (1 mL) of the spiked extract were diluted with PBS, passed through the IAC and then purified by a washing step with 6 mL of 10%, 30% and 50% MeOH. By using 10% or 30% MeOH as the washing solvent, DA recoveries for scallop (*Patinopecten yessoensis*), oyster (*Ostrea rivularis Gould*), mussel (*Mytilus edulis*), clam (*Scapharca subcrenata*) remained at 90 ± 7% or 90% ± 9%, 60 ± 3% or 65% ± 6%, 51 ± 8% or 52% ± 4%, 79 ± 2% or 71% ± 6%, respectively. For the above-mentioned samples washed by 50% MeOH, the DA recoveries were greatly improved, reaching 92 ± 7%, 91 ± 4%, 88 ± 5%, 93 ± 2%, respectively. Therefore, to maximize the elimination effect of interfering matrix components from the real shellfish extract, 6 mL of 50% MeOH was selected as the proper washing solvent.

### 2.8. Method Validation

The matrix effect for DA was evaluated based on the post-extraction addition method by comparing the peak area of a neat standard solution with that of a pretreated sample extract spiked with an equivalent amount of analyte. Experiments were carried out using scallop, oyster, mussel and clam spiked at two concentration levels (0.25 and 2.5 µg·g^−1^) as the representative matrices. Matrix effects below 8% were obtained for all the shellfish samples, indicating no significant ion suppression or enhancement observed using the developed method.

Considering the fact that mussel is the main economic shellfish in the local marine aquaculture industry and mussel homogenate exhibited most serious matrix effect for MS detection during previous IAC optimization, the method was further validated by using mussel as the representative matrix. Validation parameters included the linear range, correlation coefficient, limit of detection (LOD), limit of quantitation (LOQ), intra-day and inter-day precisions and repeatability. A calibration curve was constructed with a concentration of 0.005–4 µg·mL^−1^ DA standard solution (namely, 0.05–40 µg·g^−1^ for mussel sample), and the linearity was good with correlation coefficients (R^2^) higher than 0.998. 

LC-UV is the usual instrumental method that allows detection of DA at the concentration level of the current regulatory limit with the need for further confirmatory analysis [15]. Studies involving trace analysis of DA in shellfish and early detection of DA toxicity events usually imply additional analytical methods with higher sensitivity and qualitative function. From the method performance of higher-level spiked samples, it was possible to accurately determine the LOD and LOQ for DA in mussel tissue. Repeated analysis of 0.05 µg·g^−1^ and lower spiked samples were carried out to see if the S/N value of quantitative transition *m*/*z* 312.2 > 266.2 could be measured. The LODs (S/N = 3) and LOQs (S/N = 10) for each shellfish matrix were then found to be comparable, reaching respective values of 0.02 and 0.05 µg·g^−1^ with a 2 µL injection volume. 

**Figure 6:**
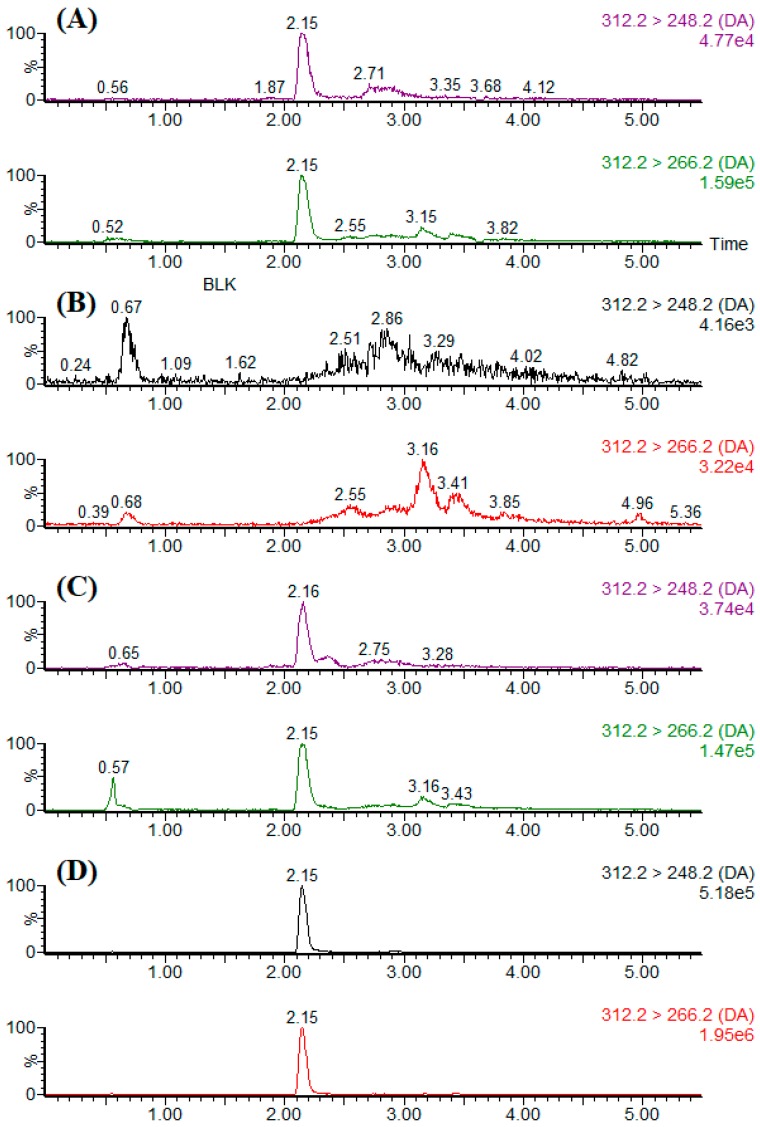
Representative MRM chromatogram of DA obtained from (**A**) 25.0 ng·mL^−1^ of standard solution; (**B**) blank mussel sample; (**C**) mussel sample spiked at a concentration of 0.25 µg·g^−1^; (**D**) naturally DA-contaminated shellfish sample.

Recoveries were performed by the standard addition method. Experiments were carried out over 5 days with a DA-free mussel homogenate spiked at three different levels (0.25, 2.5 and 25 µg·g^−1^) to assess accuracy, intra-assay and inter-assay precision (Table 3). DA recoveries for spiked samples ranged from 91–94% with intra-day RSDs of 6–8% and inter-day RSDs of 3–6%. Using the certified reference material CRM-ASP-Mus-d (49 ± 3 µg·g^−1^), recoveries of DA were 91 ± 7% (*n* = 3). Typical LC-MS/MS chromatograms of the blank sample, standard solution, spiked sample, and naturally DA-contaminated sample are shown in Figure 6.

### 2.9. Application to Real Samples

For live and raw bivalve molluscs, Codex Stan 292-2008 stipulates that the minimum applicable range, LOD and LOQ for DA determination method should meet the respective criteria of 14–26 µg·g^−1^, 2 µg·g^−1^ and 4 µg·g^−1^ [35]. When the traditional method employs SAX as the clean-up strategy, matrix-matched standards should usually be constructed for all the tested samples for accurate quantitation [20,36]. Serving as simpler and more efficient clean-up procedures, SPE deformations such as IAC, MISPE and MSPE have gained an increasing amount of attention for DA analysis in shellfish samples. In Table 4, a comprehensive comparison of the proposed method with other existing methods for DA determination was summarized in term of extraction, clean-up, LOD, linear range and recovery. Zhang et al [22] proposed an MSPE method for LC-MS/MS analysis of DA in shellfish samples by synthesizing Fe_3_O_4_·SiO_2_·UiO-66 magnetic microspheres, and Regueiro et al [37] utilized online coupling of weak anion exchange SPE and LC-UV-MS/MS to realize sensitive determination of DA in shellfish. Though LODs of the analyte in these two studies were reported at the level of pg·g^−1^ and sub ng·g^−1^, respectively, linear ranges constructed in the corresponding methods both fell outside of the one regulated by Codex Stan 292-2008. Not only were the recovery of IAC and detection limit for DA in this work comparable to other relevant reports involving deformed SPE procedures, but the performances of the developed method fulfilled the stipulation in Codex Stan 292-2008.

A total of 59 shellfish samples collected from five eastern coastal provinces between April and November 2017 were analyzed for the presence of DA. The detected species included mussel (*Mytilus edulis*), ark shell (*Scapharca subcrenata*), blood clam (*Tegillarca granosa*), different types of scallops (*Patinopecten yessoensis*, *Chlamys farreri*, *Argopecten irradians*) and oyster (*Ostrea rivularis Gould*, *Ostrea gigas Thunberg*) from local markets and culturing farms. DA has been detected in a number of different marine bivalves in the East and South China Seas, with inconsistent detection rate between 0–82% and a contaminated content of 0.02–18.2 µg·g^−1^ [7,30]. In this survey, the detection rate for DA reached 10%. Among the analyzed samples (Table 5), the largest percentage of toxic samples was from zhikong scallop (*Chlamys farreri*). Two positive zhikong scallops from the local market contained the highest DA concentrations of 14.9 and 11.8 µg·g^−1^, while the locally cultured one was found to have a low value of 0.7 µg·g^−1^. Other contaminated samples included two bay scallops (*Argopecten irradians*) from Liaoning province and one jinjiang oyster (*Ostrea rivularis Gould*) from Guangdong province, ranging from 0.1–9.3 µg·g^−1^. In all the analyzed shellfish, the DA appeared to be present at a level below the regulatory limit (20 µg·g^−1^ in edible tissue), but the results obtained in this study show a warning that the toxin still needs to be continuously monitored in the investigated bivalves, especially the ones from northeast costal area of China.

## 3. Conclusions

In this study, a highly efficient and selective method for the determination of DA in shellfish samples was developed based on IAC purification prior to UHPLC-MS/MS analysis. Clean-up procedures including loading, eluting and washing conditions on a self-assembled IAC were systematically optimized, which allowed accurate and sensitive DA quantitation without a significant matrix effect. The LOD, LOQ and applicable range of this method were 0.02 µg·g^−1^, 0.05 µg·g^−1^ and 0.05–40 µg·g^−1^, respectively. DA recoveries ranged from 91–94% with intra-day RSDs of 6–8% and inter-day RSDs of 3–6%. Method performances in this work fulfilled the regulation in Codex Stan 292-2008 and were comparable to other relevant reports. In a limited DA survey, various shellfish samples were successfully analyzed, and zhikong scallop (*Chlamys farreri*) was found to be the most toxic species. Results for method validation and application indicated that this IAC has a promising prospect for further development and commercialization as a pretreatment kit.

## 4. Materials and Methods

### 4.1. Chemicals and Reagents

A high-purity standard solution (purity higher than 98%) containing 103.3 ± 2.5 µg·mL^−1^ DA and certified reference material containing 49 ± 3 µg·g^−1^ DA (CRM-ASP-Mus-d) were purchased from the Canadian National Research Council (Halifax, Canada). Methanol (MeOH), acetonitrile (ACN), ammonium acetate and formic acid of LC grade were purchased from Sigma-Aldrich (St. Louis, MO, USA). Ultrapure water used in the preparation of reagent solutions and the mobile phase was obtained from a Milli-Q water purification system (Millipore, Bedford, MA, USA). All the other chemicals and solvents were of analytical reagent grade and were obtained from Shanghai Chemical Reagent Co. (Shanghai, China).

### 4.2. Standard Solutions and Buffers

DA stock standard solution (25 μg·mL^−1^) was prepared by dilution in MeOH and stored at 4 °C. The working standard solutions were prepared by 2 or 5 volumetric serial dilutions of the stock solution using ultrapure water containing 0.1% (*v*/*v*) formic acid and 2 mM ammonium acetate/acetonitrile (9:1, *v*/*v*) as the diluent. Phosphate buffered saline (PBS, pH 7.4) was prepared by dissolving 1.09 g of KH_2_PO_4_·2 H_2_O, 6.45 g of Na_2_HPO_4_·12 H_2_O and 4.25 g of NaCl in 500 mL of ultrapure water.

### 4.3. Preparation of Immunoaffinity Column

Cynogen bromide (CNBr) or N-hydroxy-succinimide (NHS) activated Sepharose 4B powder (1 g) was swelled within hydrochloric acid (HCl, 1 M, 200 mL) and poured into a sintered glass funnel (40–60 mm). The gel was washed with the coupling buffer (0.1 M NaHCO_3_, 0.5 M NaCl, pH 8.3, 1 L), and a 3 mL aliquot was coupled with DA monoclonal antibody (10 mg Mab mL^−1^ dissolved in the coupling buffer, 3 mL) in a stoppered flask and incubated on a shaker (120 rpm) at room temperature for 2 h. Subsequently, the mixture was washed with the coupling buffer (60 mL) to remove the free Mabs. The eluant was collected to determine the antibody amount by the Bradford protein assay method and to calculate the coupling efficiency. The unreacted active groups on the sorbents were capped with the blocking buffer (0.1 M Tris-HCl, pH 8.0) at room temperature for 2 h, then washed with 0.1 M acetate buffer (containing 0.5 M NaCl, pH 4.0) and 0.1 M Tris-HCl buffer (containing 0.5 M NaCl, pH 8.0). Finally, the Mab-coupled gel was equilibrated with 3 mL of PBS (0.01 M, pH 7.4), and then a 1 mL aliquot was transferred to a 3 mL SPE column and stored in PBS containing 0.01% (*w*/*v*) sodium azide at 4 °C.

### 4.4. Determination of Column Capacity

A relatively large amount (8 μg) of DA was spiked in PBS (3 mL) containing 25% MeOH. The solutions were then loaded into the IAC column (preconditioned with 3 mL of PBS) at a constant flow rate of 2 mL·min^−1^. The saturated column was washed with 6 mL of aqueous 50% MeOH and then eluted with 3 mL of MeOH containing 2% ammonium hydroxide. The eluate was evaporated to dryness under a stream of nitrogen at 40 °C in a water bath. The residue was reconstituted by 1 mL of ultrapure water containing 0.1% (*v*/*v*) formic acid and 2 mM ammonium acetate/acetonitrile (9:1, *v*/*v*, 1 mL) and filtered through a 0.22 μm PTFE filter into an autosampler vial for later analysis.

### 4.5. Sample Preparation

Homogenized shellfish tissue (2.00 ± 0.01 g) was weighed into a 50 mL polypropylene centrifuge tube. Then, 8 mL of aqueous 75% MeOH was added. The mixture was vortexed for 90 s, ultrasonicated for 10 min, and centrifugated at 6000× *g* for 5 min. The resulting supernatant was transferred into a new tube and the pellets re-extracted as described above. The volume of the combined supernatants was adjusted to 20 mL with the extracting solvent, and 1 mL of the sample extract was diluted with PBS at a ratio of 1:2 to adjust the final MEOH concentration to 25%.

The IAC column was preconditioned with PBS (3 mL) prior to sample loading. The analyte was loaded onto the IAC, washed with 6 mL of aqueous 50% MeOH, and then eluted with 3 mL of MeOH containing 2% ammonium hydroxide at a constant flow rate of 2 mL·min^−1^. The eluate was evaporated to dryness under a stream of nitrogen at 40 °C in a water bath. The residue was reconstituted by 1 mL of ultrapure water containing 0.1% (*v*/*v*) formic acid and 2 mM ammonium acetate/acetonitrile (9:1, *v*/*v*, 1 mL) and filtered through a 0.22 μm PTFE filter into an autosampler vial for later analysis. The columns, after elution, were immediately regenerated by PBS (50 mL) and stored in PBS containing 0.01% (w/v) sodium azide at 4 °C for subsequent use.

### 4.6. UHPLC-MS/MS Conditions

The DA analysis was carried out on an ACQUITY UPLC system coupled with a Xevo TQ-S triple-quadrupole mass spectrometer (Waters, Milford, MA, USA). The LC separation was performed on an ACQUITY UPLC BEH C18 column (50 mm × 2.1 mm I.D., 1.7 μm particle size) at 40 °C. The injection volume was 2 μL. Acetonitrile (A) and ultrapure water containing 0.1% (*v*/*v*) formic acid and 2 mM ammonium acetate (B) were used as the mobile phases, and the flow rate was 0.2 mL·min^−1^ throughout the analysis. The chromatographic resolution for DA and its isomers was set as follows (t is time and subscript numbers are the time in minute): t_0_, B = 90; t_3_, B = 90; t_3.5_, B = 10; t_4.5_, B = 10; t_5_, B = 90; t_7_, B = 90. The gradient for the sample cleaned up by IAC was set as follows: t_0_, B = 90; t_1_, B = 90; t_3_, B = 10; t_4_, B = 10; t_4.1_, B = 90; t_5.5_, B = 90.

Analyte was detected by multiple reaction monitoring (MRM) using electrospray (ESI) positive ion mode. The MS/MS detection parameters were optimized by standard infusion and set as follows: capillary voltage, 3.0 kV; source temperature, 150 °C; desolvation temperature, 600 °C; cone gas flow, 150 L·h^−1^; desolvation gas flow, 1000 L·h^−1^; cone voltage, 12 V; collision energy, 16 eV. The precursor/product ion *m*/*z* 312.2 > 266.2 and *m*/*z* 312.2 > 248.2 were used for quantitative determination and qualitative confirmatory, respectively.

### 4.7. Data Analysis

Data were acquired and processed using MassLynx 4.1 and QuanLynx software (Waters, Milford, MA, USA). The DA concentrations in samples (µg·g^−1^) were calculated directly from the area responses using a linear seven-point calibration ranged from 0.005–4 µg·mL^−1^. The confirmatory guidelines for DA were in agreement with the performance criteria of European Commission (EC) Decision 2002/657/EC [39]. The maximum permitted tolerances for the relative retention time and relative ion intensities (ion ratio) should be within ± 2.5% and ± 25%, respectively.

## Figures and Tables

**Table 1:** Effect of the methanol percentage and number of extractions on the recoveries of DA (*n* = 3).

DA Recovery (%)	Number of Extraction	
	1	2
50% MeOH	82 ± 8	99 ± 8
75% MeOH	88 ± 3	102 ± 6
90% MeOH	71 ± 6	81 ± 7

**Table 2:** Recoveries of DA under different eluting conditions (*n* = 3).

Elution Fraction	Recovery in Various Fractions (%)		
	1% Formic Acid in MeOH	1% Ammonium Hydroxide in MeOH	2% Ammonium Hydroxide in MeOH
fraction 1 (1 mL)	83	69	77
fraction 2 (1 mL)	4	15	16
fraction 3 (1 mL)	ND ^b^	2	2
fraction 4 (1 mL)	ND	2	ND
fraction 5 (1 mL)	ND	ND	ND
total recovery ^a^	87	88	95

^a^ Total recovery is the sum of fraction 1 to fraction 5. ^b^ ND indicates not detected.

**Table 3:** Accuracy, intra-assay and inter-assay precision of the developed method using a blank mussel spiked at three different levels.

Spiked Level (µg·g^−1^)	Intra-Day RSD (%, *n* = 5)	Inter-Day RSD (%, *n* = 3)	Recovery ± SD (%, *n* = 5)
0.25	7	6	91 ± 3
2.5	6	3	94 ± 5
25	8	5	92 ± 6

**Table 4:** A comparison between previously reported methods and the proposed method for LC determination of DA in shellfish.

Instrumental Method	Mode	Extraction	Clean-Up	LOD	Linear Range	Recovery	Reference
LC-UV	Offline	HCl	immunoaffinity column	- ^d^	-	85–90%	[21]
LC-MS/MS	Offline	Aqueous MeOH	Strong anion SPE	0.014 µg·mL^−1^	0.025–10 µg·mL^−1^	92%	[18]
LC-UV	Offline	Aqueous MeOH	MIP ^a^ SPE	0.1 µg·mL^−1^	0.5–25 µg·mL^−1^	93–97%	[9]
LC-UV	Online	Aqueous MeOH	MIP monolithic column	0.076 µg·mL^−1^	-	89–91%	[38]
LC-MS/MS	Offline	Aqueous MeOH	Fe_3_O_4_·SiO_2_·UiO-66 MSPE ^b^	1.45 pg·mL^−1^	2–1000 pg·mL^−1^	93–107%	[22]
LC-UV-MS/MS	Online	Aqueous MeOH	weak anion SPE	0.3 ng·g^−1^ (MS/MS) 4–10 ng·g^−1^ (UV)	0.05–100 ng·mL^−1^ (MS/MS) 0.25–200 ng·mL^−1^ (UV)	94–102%	[37]
LC-MS/MS	Offline	methanol/acetone	Florisil (Extraction with PLE ^c^)	0.2 µg·g^−1^	0.05–5 µg·mL^−1^	81–95%	[20]
LC-MS/MS	Offline	Aqueous MeOH	immunoaffinity column	0.02 µg·g^−1^	0.05–40 µg·g^−1^	91–94%	This work

^a^ MIP indicates molecularly imprinted polymer. ^b^ MSPE indicates magnetic solid-phase extraction. ^c^ PLE indicates pressurised liquid extraction. ^d^ - indicates not mentioned.

**Table 5:** DA Concentrations (µg·g^−1^) in 59 shellfish samples collected from eastern coastal provinces in China between April and November 2017.

Species	Total Number of Samples Analyzed	No. Positive Samples	Sampling Location	Sampling Time	DA Level (µg·g^−1^)
yesso scallop (*Patinopecten yessoensis*)	4	0			ND ^a^
zhikong scallop (*Chlamys farreri*)	12	3			
		No. 1	Huludiao, Liaoning/local market	September 2017	11.8
		No. 2	Huludiao, Liaoning/local market	September 2017	14.9
		No. 3	Xingcheng, Liaoning/culturing farm	September 2017	0.7
bay scallop (*Argopecten irradians*)	10	2			
		No. 1	Huludiao, Liaoning/local market	September 2017	9.3
		No. 2	Xingcheng, Liaoning/culturing farm	September 2017	3.2
jinjiang oyster (*Ostrea rivularis Gould*)	9	1			
		No. 1	Shantou, Guangdong/culturing farm	October 2017	0.1
long oyster (*Ostrea gigas Thunberg*)	6	0			ND
mussel (*Mytilus edulis*)	8	0			ND
ark shell (*Scapharca subcrenata*)	5	0			ND
blood clam (*Tegillarca granosa*)	5	0			ND

^a^ ND indicates not detected.
